# Hypertrophic obstructive cardiomyopathy-left ventricular outflow tract shapes and their hemodynamic influences applying CMR

**DOI:** 10.1007/s10554-024-03242-4

**Published:** 2024-09-20

**Authors:** T. Mayr, L. Riazy, R. F. Trauzeddel, J. P. Bassenge, S. Wiesemann, E. Blaszczyk, M. Prothmann, T. Hadler, S. Schmitter, Jeanette Schulz-Menger

**Affiliations:** 1 ECRC Experimental and Clinical Research Center, Charité – Universitätsmedizin Berlin, Corporate Member of Freie Universität Berlin and Humboldt-Universität zu Berlin, Lindenberger Weg 80, 13125 Berlin, Germany; 2 Working Group on Cardiovascular Magnetic Resonance, Experimental and Clinical Research Center, Charité Medical Faculty and the Max-Delbrück Center for Molecular Medicine, Charité – Universitätsmedizin Berlin, Lindenberger Weg 80, 13125 Berlin, Germany; 3 Partner Site Berlin, DZHK (German Centre for Cardiovascular Research), Berlin, Germany; 4 Physikalisch-Technische Bundesanstalt (PTB), Braunschweig and Berlin, Berlin, Germany; 5 HELIOS Hospital Berlin-Buch, Berlin, Germany; 6 Department of Anesthesiology and Intensive Care Medicine, Charité - Universitätsmedizin Berlin, Corporate Member of Freie Universität and Humboldt Universität zu Berlin, Campus Benjamin Franklin, Berlin, Germany

**Keywords:** Hypertrophic cardiomyopathy, Hypertrophic obstructive cardiomyopathy, Cardiovascular magnetic resonance, Left ventricular outflow obstruction, Hemodynamics, Shape analysis

## Abstract

Hypertrophic cardiomyopathy (HCM) is one of the most common genetic cardiac disorders and is characterized by different phenotypes of left ventricular hypertrophy with and without obstruction. The effects of left ventricular outflow tract (LVOT) obstruction based on different anatomies may be hemodynamically relevant and influence therapeutic decision making. Cardiovascular magnetic resonance (CMR) provides anatomical information. We aimed to identify different shapes of LVOT-obstruction using Cardiovascular Magnetic Resonance (CMR). The study consisted of two parts: An in-vivo experiment for shape analysis and in-vitro part for the assessment of its hemodynamic consequences. In-vivo a 3D depiction of the LVOT was created using a 3D multi-slice reconstruction from 2D-slices (full coverage cine stack with 7 slices and a thickness of 5–6 mm with no gap) in 125 consecutive HOCM patients (age = 64.17 +/− 12.655; female *n* = 42). In-vitro an analysis of the LVOT regarding shape and flow behavior was conducted. For this purpose, 2D and 4D measurements were performed on 3D printed phantoms which were based on the anatomical characteristics of the in-vivo study, retrospectively. The in-vivo study identified three main shapes named K- (28.8%), X- (51.2%) and V-shape (10.4%) and a mixed one (9.6%). By analyzing the in-vitro flow measurements every shape showed an individual flow profile in relation to the maximum velocity in cm/s. Here, the V-shape showed the highest value of velocity (max. 138.87 cm/s). The X-shape was characterized by a similar profile but with lower velocity values (max. 125.39 cm/s), whereas the K-shape had an increase of the velocity without decrease (max. 137.11 cm/s). For the first time three different shapes of LVOT-obstruction could be identified. These variants seem to affect the hemodynamics in HOCM.

**Supplementary Information:**

The online version contains supplementary material available at 10.1007/s10554-024-03242-4.

## Introduction

Hypertrophic cardiomyopathy (HCM) is known to be one of the most common occurring genetic cardiac disorders with a prevalence of 1:500 [1]. It is characterized by different phenotypes of left ventricular hypertrophy [2–4]. Several subtypes of HCM show a morphology which may affect the left ventricular outflow tract (LVOT) and can induce a hemodynamic relevant obstruction, which occurs in 70% of the HCM-patients [5]. Various morphologies of the mitral valve [2, 3, 6, 7] as well as the papillary muscles [7, 8] further influence the severity and localization of such obstruction [1, 2, 9]. The symptoms of HCM vary greatly, ranging from asymptomatic to dyspnea, angina pectoris, arrhythmias, and syncope [8, 10–12]. The most feared complication is sudden cardiac death, particularly in young athletic patients [8]. In terms of risk stratification for this, syncope or pre-syncope are particularly noteworthy. It is known that patients with an obstructive component are more prone to syncope and are therefore at higher risk [11]. Consequently, we believe that a closer examination of the LVOT can provide additional information that may influence and potentially simplify therapeutic decision making. By characterizing patients with obstruction according to its shape and thus according to the severity of the obstruction, earlier decisions for invasive therapy could be made to reduce the risk of syncope. Especially in invasive methods, anatomy plays a significant role [1, 8]. Regarding non-invasive therapy, management is often difficult because symptoms can vary depending on patient activity or changes in cardiac volume. In the literature, the LVOT is described only in terms of its pure anatomy. There is no concrete categorization of the entire outflow tract. In clinical routine 2D or 3D echocardiography is used to depict the anatomy and diagnose HCM. To quantify the obstructive subtype the simplified Bernoulli formula is used for calculating the pressure gradient in an indirect way [1–3, 6, 10]. Advantages of echocardiography are that it is noninvasive, fast and universally available. However, it is very dependent on the examiner and may be hindered by an impaired ultrasound window [13]. The direct way to measure an obstruction is to use a cardiac catheter. It is more accurate but also much more invasive and not suitable for describing the anatomy [3, 6, 11, 12, 23]. Cardiovascular magnetic resonance (CMR) is capable of independently and precisely depicting the anatomy. It is also possible to conduct a comprehensive non-invasive flow study, for example, with three-dimensional (3D) cine (time resolved) phase-contrast CMR with three-directional velocity encoding (4D Flow CMR). Thus, it combines the advantages of echocardiography and invasive flow analysis into a widely used non-invasive procedure [4, 9, 13, 14].

Both such an analysis of patients anatomy and calculation of obstruction degree are termed prerequisites in making optimal therapeutic decisions.

One could assume that different patterns of septal wall and mitral valve anatomy may lead to different types of obstruction and may consequently require different therapeutic strategies. To our knowledge, there are no LVOT characterizations described in literature.

Therefore, the aim of this study was to identify different shapes of the LVOT and evaluate their hemodynamic implications.

## Methods

The study consisted of two parts: An in-vivo experiment for shape analysis and an in-vitro part for the assessment of its hemodynamic implications.

In-vivo, we focused on the assessment and categorization of LVOT shapes based on CMR cine-images in HCM patients.

In-vitro, a 3D phantom study, reflecting the identified LVOT obstruction patterns to further examine the hemodynamic implications of these obstruction forms using phase-contrast based two-dimensional blood flow CMR (2D Flow CMR) and 4D Flow CMR was used.

**Fig. 1:**
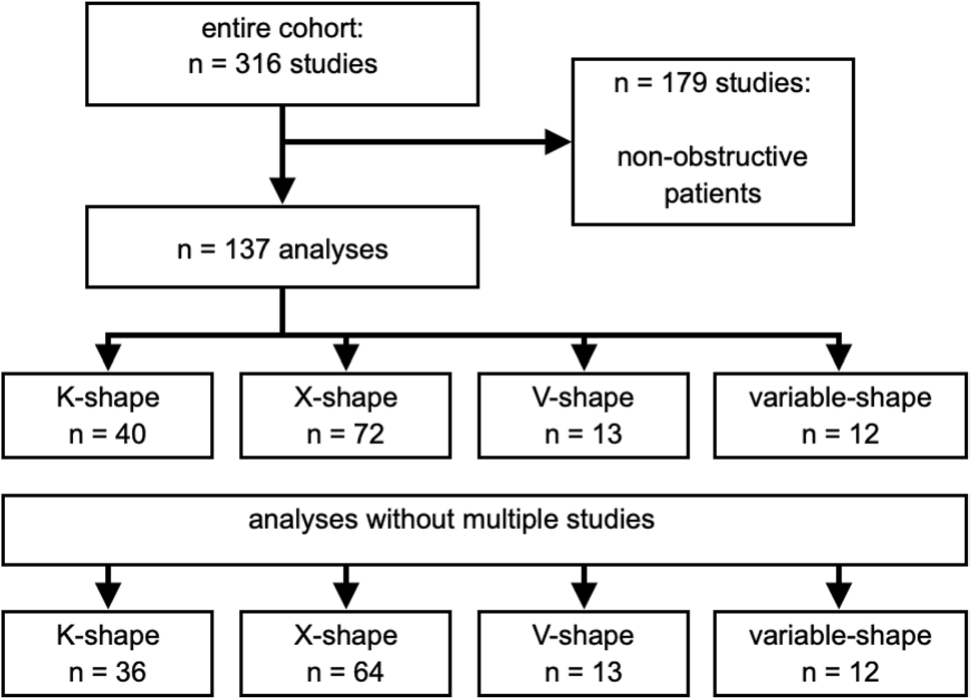
Study cohort with the results of the shape differentiation

The first author of the manuscript has full access to all data used and takes responsibility for the integrity and analyses (Fig. 1).

## In-vivo study

The CMR scans were identified by screening our database from 2007 to 2018. Inclusion criteria were a clinically diagnosed HCM and a CMR scan with full coverage of the LVOT. Exclusion criteria were age < 18 years, no existing clinical diagnosis of HOCM, rhythm disorders such as atrial fibrillation, general contraindications for CMR scans and lack of coverage of the LVOT in the CMR measurements. Consecutively, scanned HCM patients with hypertrophic obstructive cardiomyopathy (HOCM) could be included and were retrospectively analyzed. The study was approved by the ethical committee of the Charité – Universitätsmedizin Berlin (EA1/076/18).

## CMR

The CMR scans were performed using either a 1.5 Tesla (T) (Avanto and Avanto fit, Siemens Healthineers, Erlangen, Germany) or a 3 T (Verio, Siemens Healthineers, Erlangen, Germany) scanner. All scan protocols were similar and included multi-slice full coverage of the LVOT using cine-SSFP with a slice thickness of 5 to 6 mm with no gap and an acquisition matrix of 192 × 125 mm – 192 × 174 mm as published previously [9].

## Postprocessing

LV-enddiastolic and -endsystolic volume, myocardial mass and papillary muscle were quantified. Furthermore, ejection fraction as well as stroke volume were calculated in a standardized procedure using Cvi42 Version 5.6.6 (Circle Cardiovascular Imaging Inc., Calgary, Canada) [15].

**Fig. 2:**
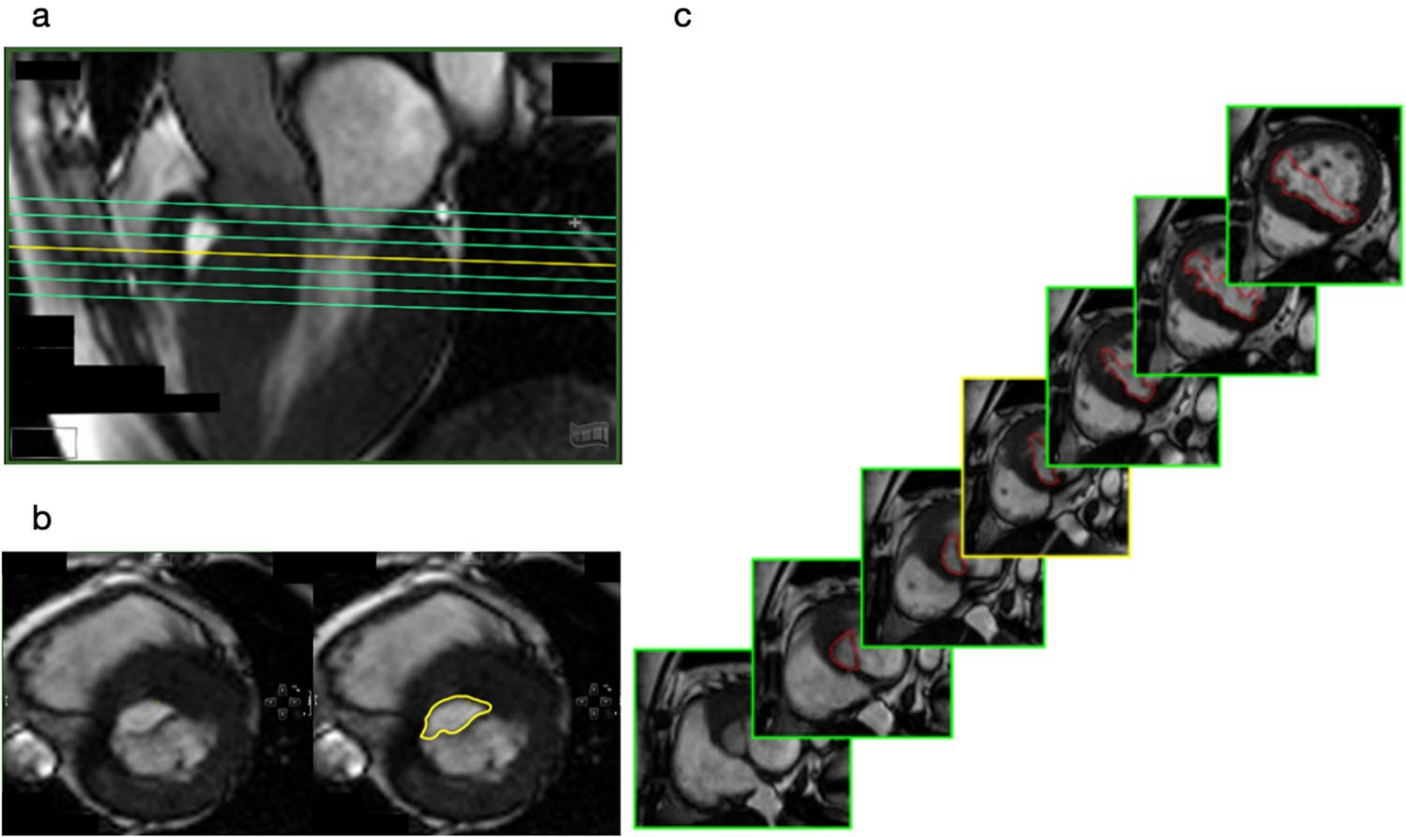
**(a)** short axis package (SAX) covering the entire LVOT with seven slices; **(b)** Cine images showing the LVOT without (left) and with contours (right); **(c)** exemplary short axis cross sections with marked LVOT

**Fig. 3:**
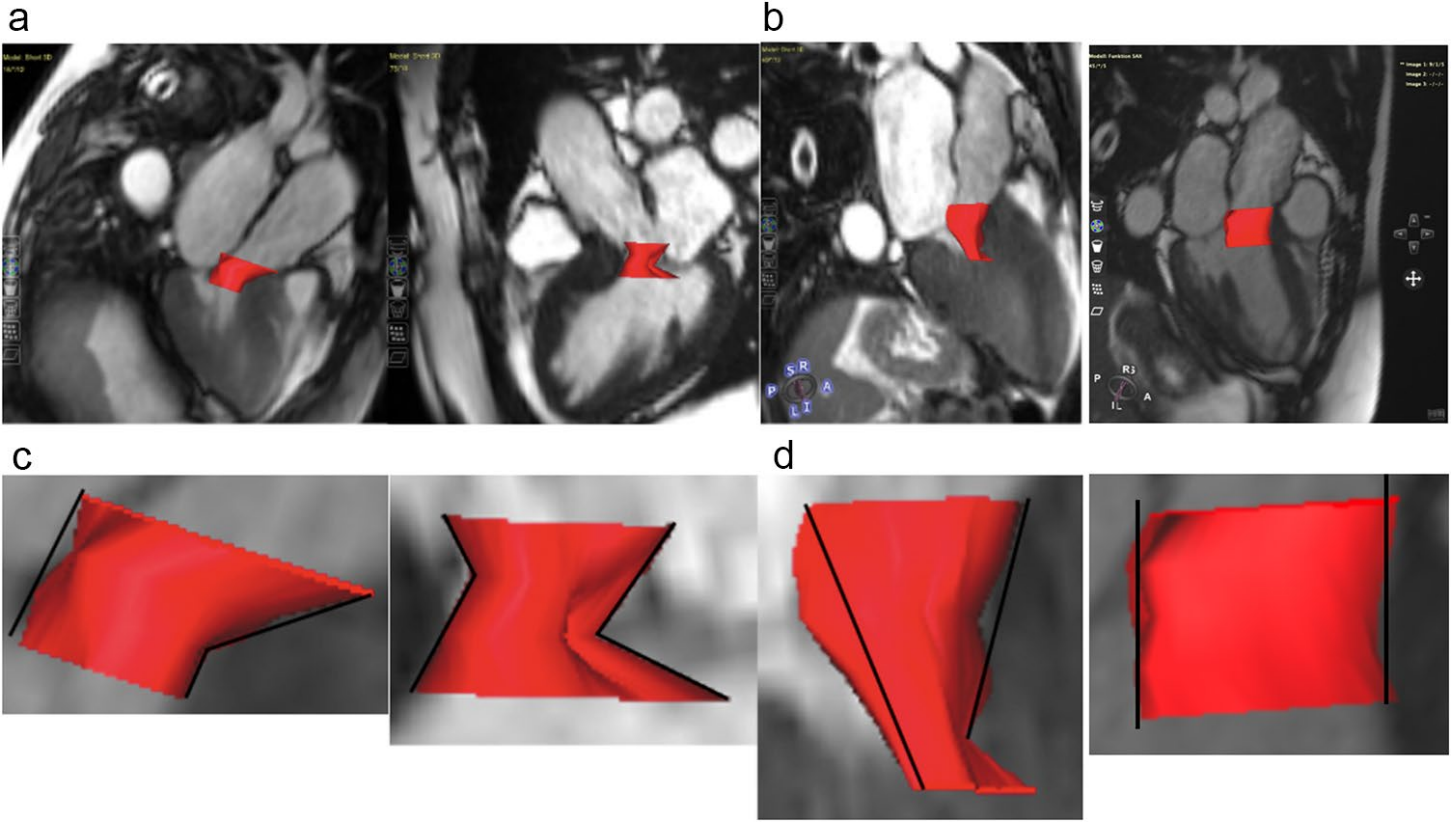
a-c three chamber view of the different shapes; **(a)** K-shape; **(b)** X-shape; **(c)** V-shape; **(d)** O-shape

The LVOT-analysis primarily involved the identification of shapes and was based on the quantification of a multi-slice stack covering the whole LVOT. The contouring of the LVOT-area was performed in every phase and slice (Fig. 2) allowing for a 3D multislice reconstruction of the LVOT. The 3D reconstruction generated could now be aligned to all planes within the Cvi42 workspace. For consistency in evaluation, we chose to align it in the longitudinal view at the timepoint of end-sytsole. Anatomically, this means viewing the LVOT with the aortic ring at the top, the septum on the left, and the mitral valve on the right. Four different shapes could be identified, three of them being distinct and one mixed shape. Following their pattern, the distinct main shapes were named K-, X- and V-shape (Fig. 3 a–c). The shapes were defined based on a comparison made to a 3D LVOT shape of healthy volunteers.

The K-shape was characterized by a unilateral volume reduction between the upper and middle third of the LVOT induced by hypertrophy of the interventricular septum (Fig. 3a). The X-shape showed a bilateral volume reduction between the middle and lower third of the LVOT induced by hypertrophy of the interventricular septum and by the systolic anterior movement of the mitral valve (SAM) as seen in Fig. 3b.

The V-shape was also formed by a bilateral reduction of the LVOT volume. However, in contrast to the X-shape, the obstructive area was located between the tip of the anterior valve leaflet and the basal part of the septal wall (Fig. 3c).

**Fig. 4:**
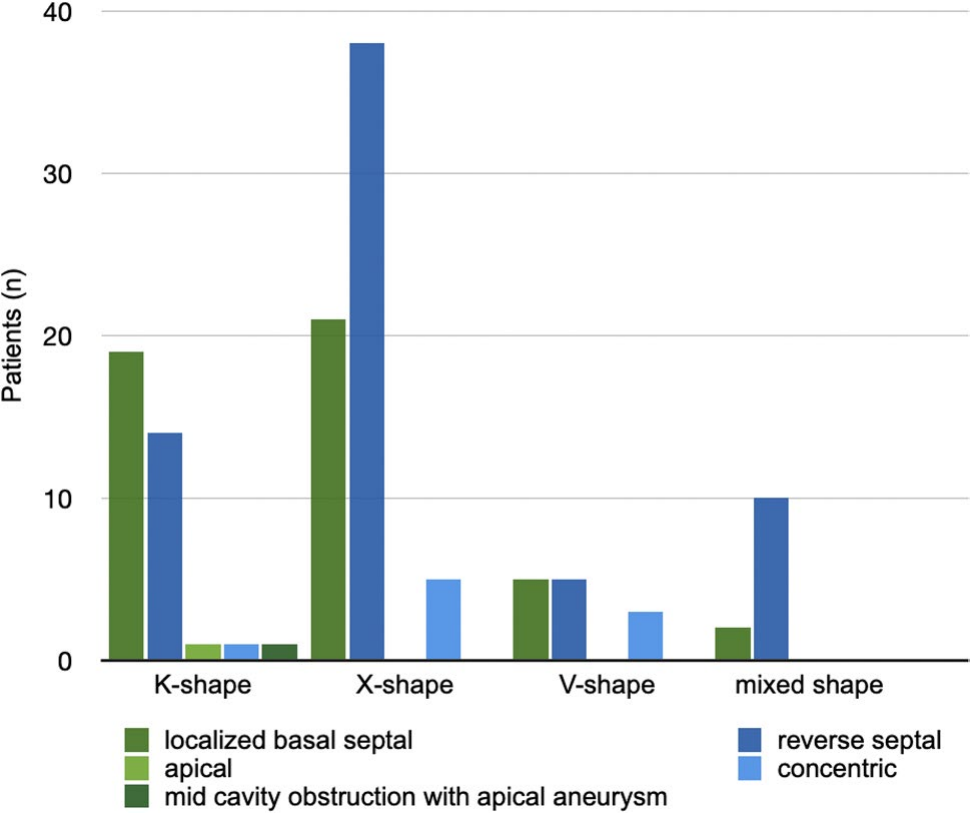
Distribution of hypertrophy types by shape of obstruction

In Fig. 4, we have presented a comparison between the individual shapes and the locations of the hypertrophies.

A narrowing was defined as 1/8 of the average because it was the best discriminator between the shapes. This value was found out by testing the lowest common denominator for an obstruction in a probatory way.

The mixed-shape was characterized by changing size and shape during systole. So in this case there wasn’t the opportunity to make a clear decision. In contrast, the three main shapes became clearer in their morphology from the start of the systole to the endsystolic timepoint.

Thirty patients each were randomly selected for testing intra- and interobserver variability using Kohen´s kappa. The analysis was performed by a SCMR Level III reader.

## In-vitro study

**Fig. 5:**
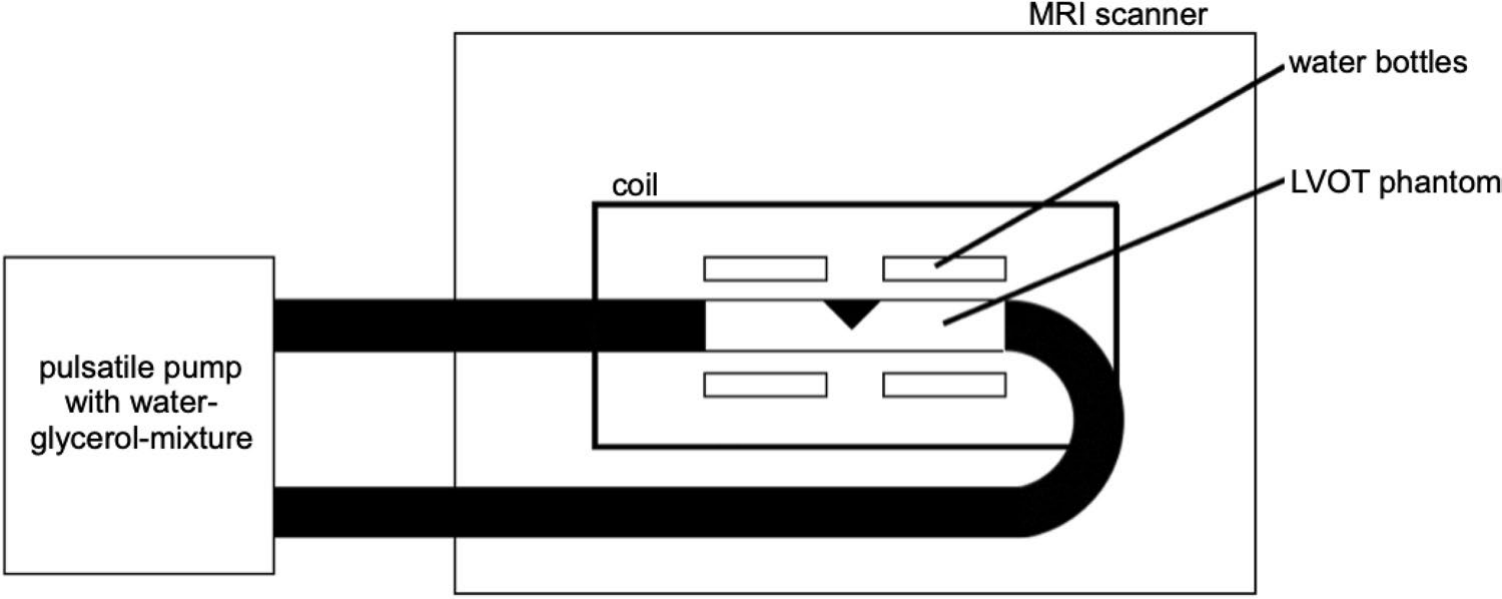
outlined experimental setup of the phantom circuit

**Fig. 6:**
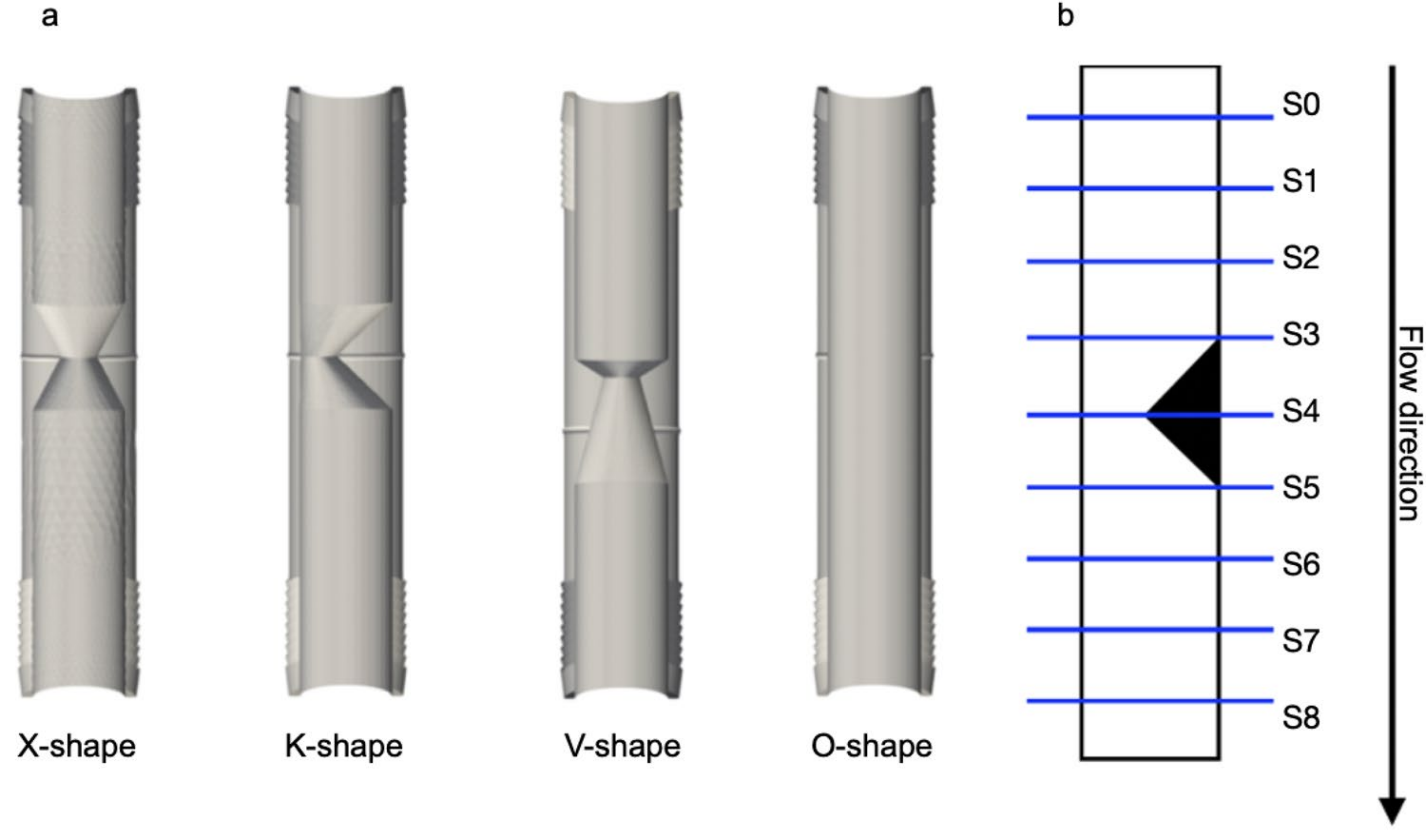
**(a)** 3D LVOT phantoms; control = “healthy” O-shape; **(b)** schematic illustration of the LVOT with the set-up of segmentation

The different LVOT-shapes were printed as idealized 3D models using a resin material (Contura Modellbau Berlin, Germany). The area of narrowing was equal in all three phantom models. This is a magnitude value that was obtained from in-vivo measurements and then normalized for all three shapes. For the comparison of the three obstructive shapes we used a straight through tube as a “healthy” LVOT in the phantom to simulate unobstructed flow. The experimental setup (Figs. 5 and 6) consisted of a closed flexible tube system with an inner diameter of 32 mm and a total length of 9.5 m, as well as the pulsatile pump Cardio Flow 5000 MR (Shelley Medical Imaging Technologies, Toronto, Ontario, Canada). The flow was adjusted to a velocity of 200 ml/s. To simulate the viscosity of blood a room-temperature mixture consisting of 60% distilled water and 40% glycerol [12] was used, which was filled into a reservoir on the pump at the end of the scanner table.

The scans took place on a 3T MR-scanner (Verio, Siemens Healthineers, Erlangen, Germany). Flow measurements were performed using a standard 2D Flow CMR sequence (field of view (FOV) = 128 × 48 mm; resolution = (1.0 mm)² x 5 mm; VENC = 2.0 m/s; TR = 5.73 msec; retrospective). The 4D Flow CMR sequence had the following parameters (FOV = 192 mm x 48 mm x 48 mm; resolution (1.0 mm)^3^; VENC = 2.0 m/s; TR = 7.70 msec.)

Nine slices (S0-S8) were positioned perpendicular to the direction of flow across the whole length of the phantom: three planes before, three inside and three planes after the area of obstruction (Fig. 6).

The maximum velocity was analyzed using Cvi42 (Circle Cardiovascular Imaging Inc., Calgary, Canada, Version 5.6.6), whereas a prototype of the same company (4D Prototype Version 5.13.0) served for 4D Flow CMR assessment. The segmentation was done manually in nine predefined planes as shown in Fig. 6b with planes S3–S5 corresponding with the obstruction. A correction of noise and velocity aliasing was made automatically by Cvi42. Velocity-encoding streamlines were visually assessed according to the methodology of Allen et al. [2] using a three-level graduation. Linear flow was categorized as grade 0. Helical flows with rotation < 360° were grade 1 and flows with rotations > 360° were declared as grade 2.

**Fig. 7:**
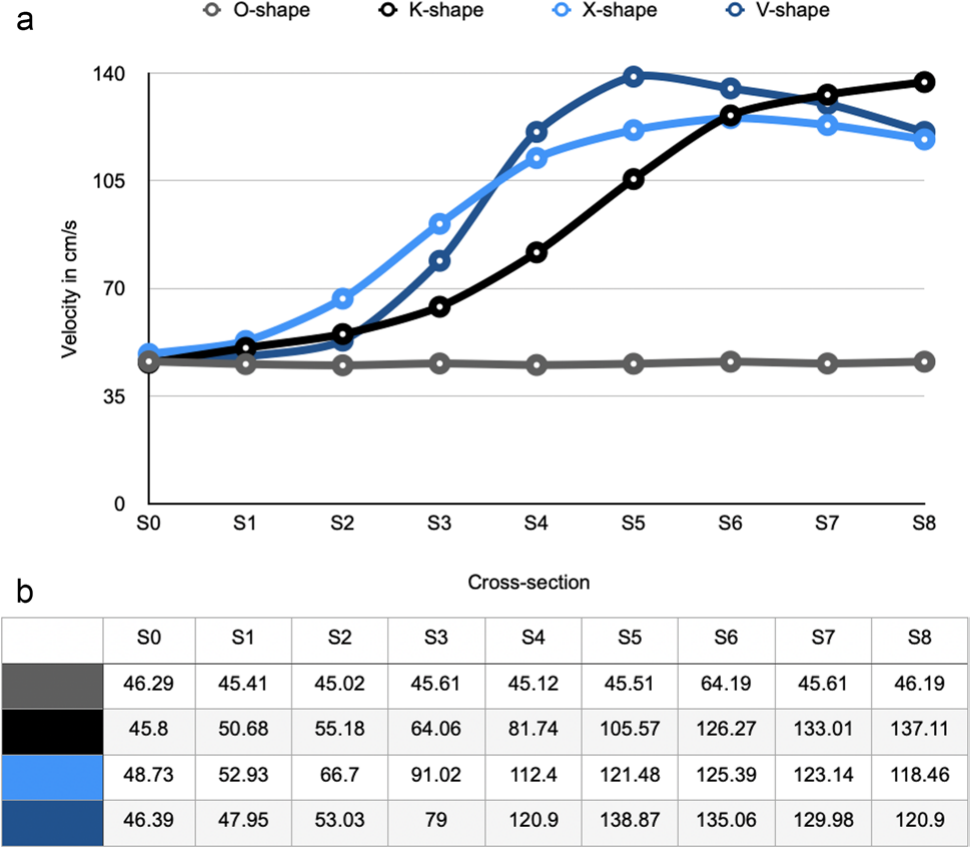
**(a)** 2D Flow CMR results with the parameter maximum velocity in cm/s in every single slice; **(b)** Velocity values per cross section categorized by the four different shapes

The results were compared to 2D Flow CMR results in the same planes adjusted to the maximum systolic accelerated flow (Fig. 7).

## Statistics

For the statistical analysis, SPSS Version 26 (IBM, Armonk, New York, USA) was used. Interval-scaled data were given in mean and standard deviation (SD) values and nominal and ordinal data in absolute and percentage values. The comparison of the patient characteristics were carried out using the Mann-Whitney test. The LVOT-shapes were compared amongst each other using the Kruskal-Wallis test.

For the analysis of the intra- and interobserver testing Kohen´s kappa was applied. Statistical significance was assumed at a value of *p* < 0.05.

The programs Microsoft Office Word, Excel version 16.83 (Microsoft, Redmond, Washington) and Pages by Apple Inc. version 13.2 (Apple Inc, Cupertino, California) were used to create tables and figures.

## Results

### Patient demographics

316 HOCM patients with LVOT obstruction could be identified in our database. Of those, 179 patients had to be excluded with consideration of the inclusion criteria. Twelve of the remaining 137 patients had several MR scans (Fig. 1) with a consistent shape. These twelve were only counted once. The final analysis included 125 patients (33.6% female; age = 64.17 +/− 12.655).

The basic patient characteristics including demographics and LV characteristics were summarized in Table 1.

**Table 1:** Patient characteristics by main shapes

	K	X	V	Variable	P*
Age (years)	58.69 +/− 9.32	56.73 +/− 13.34	58.00 +/- 10.55	57.83 +/− 12.68	0.966
Sex	m = 26/f = 10	m = 44/f = 20	m = 8/f = 5	m = 5/f = 7	/
EF (%)	65.89 +/− 7.94	66.5 +/− 8.83	66.15 +/− 6.09	68.25 +/− 5.28	0.740
EDV (ml)	133.42 +/− 33.12	149.67 +/− 35.32	134.85 +/− 29.15	136.25 +/− 27.83	0.173
ESV (ml)	45.14 +/− 14.03	50.91 +/− 21.37	45.08 +/− 10.29	43.17 +/− 10.15	0.801
SV (ml)	88.28 +/− 25.23	98.77 +/− 23.92	89.77 +/− 22.21	93.08 +/− 21.08	0.241
LVEDVI (ml/m^2^)	66.7 +/− 12.65	74.47 +/− 16.36	70.24 +/− 15.25	72.85 +/− 15.60	0.225
LVESVI (ml/m^2^)	22.61 +/− 6.28	25.40 +/− 10.42	23.41 +/− 4.77	23.02 +/− 5.73	0.900
Mass myocardial (g)	168.28 +/− 70.89	201.27 +/− 73.76	185.77 +/− 65.63	175.92 +/− 48.76	0.118
LVMI (g/m^2^)	83.36 +/− 30.5	99.94 +/− 35.07	94.94 +/− 26.21	94.45 +/− 27.93	0.052
Mass papillary muscle (g)	5.58 +/− 4.56	5.47 +/− 2.62	5.69 +/− 3.22	5.00 +/− 1.81	0.887

* *p* < 0,05 = significant; Kruskal-Wallis

Patients’ distribution in the obstructive subgroups were as follows: K-shape (*n* = 36; 28.8%), X-shape (*n* = 64; 51.2%), V-shape (*n* = 13; 10.4%) and mixed-shape (*n* = 12; 9.6%).

Intra- and interobserver analysis of LVOT-shape:

Kohen’s kappa was 0.794 for interobserver and 0.847 for intraobserver testing.

### In vitro study – analysis of hemodynamic

**Fig. 8:**
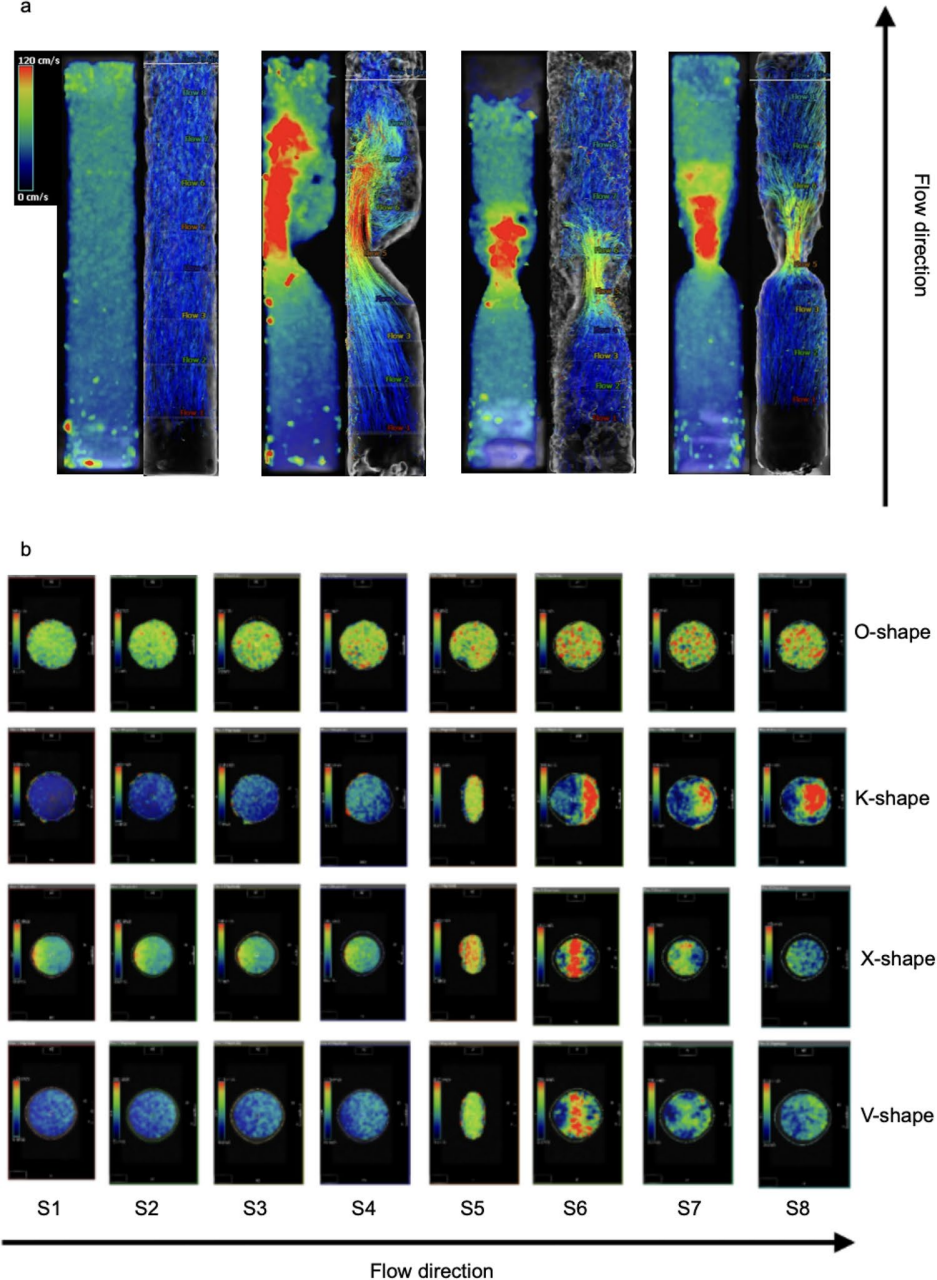
4D Flow CMR visualization of the four phantom models with the path lines which includes the cross sections of each shape next to the velocity visualizations; in order: O-shape, K-shape, X-shape, V-shape; **(b)** 4D Flow CMR patterns of the different cross sections

**Fig. 9:**
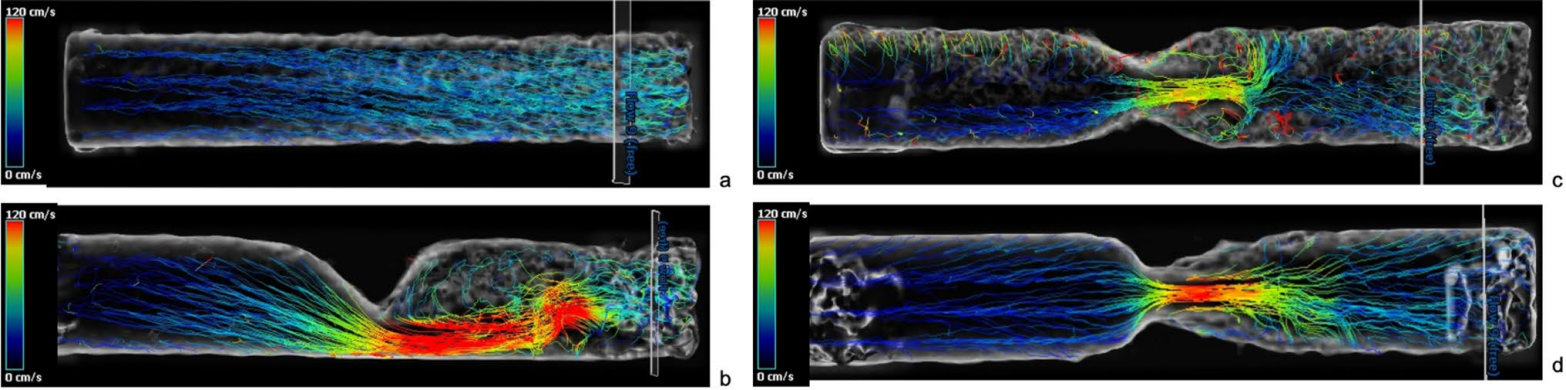
a-d 4D Flow CMR-visualization with streamlines showing the helical and vortical character of the flow; **(a)** O-shape with laminar flow corresponds with grade 0; **(b)** K-shape with helical flow corresponds with grade 2; **(c)** X-shape with helical flow grade 1; **(d)** V-shape with helical flow grade 1

The 3D phantom served for analyzing the three main types, K-shape, X-shape, V-shape and the control (O-shape). The comparison of the velocity curves in relation to the parameter maximum velocity in every slice is shown in Fig. 7. The visualizations of the 4D Flow CMR data are given in the Figs. 8 and 9. Figures 8a and 9a-d illustrate the corresponding longitudinal visualizations, whereas Fig. 8b illustrates the crossections. The three obstruction types had unique flow profiles. The K-shape was characterized by a jet flow close to the wall with a self-centering tendency and a helical flow profile downstream of the obstructive area. The X- and V-shape both showed a central jet, however the X-shape displayed higher velocity values in the beginning of the obstruction with a more scattering profile than the V-shape. Using the streamlines in Fig. 9 we were able to classify the helical characteristics of the flow following the methods of Allen et al. [2]; the K-shape shows a helical flow grade 2 whereas the X- and V-shape showed a helical flow grade 1.

The O-shape was characterized by a continuous velocity profile without evidence of any turbulent flow patterns with an average maximum velocity of 45.66 cm/s. The 2D Flow CMR patterns of the other shapes had varying velocity profiles. The maximum velocity from each plane was analyzed and compared to each other. Surprisingly, the K-shape illustrated an increase of the velocity values without any decrease within the phantom, with an absolute maximum velocity of 137.11 cm/s. The X- and the V-shape showed an increase of the values in the obstruction phase followed by a phase of decrease. The main differences were visible after the center of the obstruction, represented by the cross section S5. The peak velocity was noted as 121.48 cm/s in the X-shape and 138.87 cm/s in the V-shape, respectively. In contrast, the K-shape showed a lower velocity of 105.57 cm/s at S5. The maximum velocity in the K-shape reported was 137.11 cm/s at S8. The X-shape had the turnover point at cross section S6 with a maximum velocity of 125.39 cm/s, whereas it was directly at S5 in the V-shape with velocity reaching 138.87 cm/s.

The X- and V-shapes showed pattern similarities in the trend of flow behavior but differed in the variability of velocity changes. Both shapes started their flow profiles with a basal flow velocity near the O-shape (X-Shape 48.73 cm/s; V-shape 46.39 cm/s). Then they showed an increase to their maximum velocities followed by a trend to a decrease towards the end of the measurements with velocity of 118.46 cm/s in the X-shape and 120.9 cm/s in the V-shape.

## Discussion

The hemodynamic impact and the morphology of LVOT-obstruction in HOCM-patients is of ongoing interest and remains a challenge. In our analysis of LVOT obstructions in HOCM three main types could be identified. These shapes were characterized by different hemodynamic patterns with the V-shape displaying the most pronounced obstruction. To the best of our knowledge this is the first study describing different LVOT-obstruction shapes using a CMR based 3D reconstruction approach.

The septal hypertrophy described in all three obstructive shapes led to a unanimous unilateral reduction of volume while the bilateral obstructive narrowing was induced by different structures such as the anterior leaflet of the mitral valve or the papillary muscles. The K-shape was based solely on septal hypertrophy. The X- and V-shapes showed similarities in their formed anatomies in the septal hypertrophy and the SAM-phenomenon, however the V-shape’s distinctive influence was caused by an asymmetric thickness and the hypertrophy of the papillary muscles. This further underlines the impact of the papillary muscle size [16, 17]. In HCM the quantification of the papillary muscles as a part of the myocardium are impactful, as they influence the stroke volume as well as the obstruction. Another shape was the so-called mixed shape. This is neither a new main shape nor a shape that can be directly categorized, as it changes its external appearance during the systolic phases. Different aspects of the three main categories were all present within in this shape. Anatomically, the same features of the other categories are also present, which result in an obstructive pattern. It is speculated that this might be an intermediate form in the progression of the disease. However, we cannot confirm this precisely, as the number of patients with this type was the smallest within the cohort. On the other hand, patients who had multiple scans over several years showed consistency in the assigned category after the initial measurement. Therefore, it remains questionable how the mixed shape should be evaluated. Further analyses with new measurements would be necessary for this.

In the NHLBI HCM Registry (HCMR trial) different phenotypes of HCM could be identified. With such a vast prospective cohort, the asymmetric septal hypertrophy was shown to be the most common phenotype [4]. In our analysis of the distribution (Fig. 4), it was evident that the K- and X-shape are the most common shape of obstruction. These primarily result from a local hypertrophy of the septum, which aligns with the finding that septal hypertrophy is the most frequently occurring. This leads to a direct local impact on the LVOT-pathway and thus to altered flow patterns in the sense of an obstruction. Usually, the pressure gradient is used to quantify the obstruction. Techniques like 4D Flow CMR may provide additional information, but they are not currently applied in clinical routine in CMR despite the application in congenital heart disease. 4D Flow CMR visualization is widely used in such patients as advised in the guidelines [18]. In HCM Allen et al. [2] could show that different grades of obstructive flow profiles could be differentiated by velocity isosurfaces of the LVOT using 4D Flow CMR imaging. They have pointed out the relevance of the SAM-phenomenon by identifying a pronounced helical flow in patients with obstruction. Based on our in-vitro experiments we hypothesize that helical flow patterns causes the flow phenomenon. Our phantom provides information at the time of end-systole, which is the point of maximum obstruction. It is known that helical flow patterns increase with the severity of the obstruction. To create identical conditions among our obstruction shapes, we normalized the area of narrowing in all three phantoms. It was found that despite the identical conditions, flow patterns were different, and the intensity of the helical flow varied among the shapes. Care was taken to identify the one with the most significant obstruction. The LVOT could be compared with a nozzle inducing a jet flow [24]. These jet formations represented the three main obstructive shapes. Interestingly, even the X- and V-shapes, although very similar in shape pattern, showed different flow profiles. We can only assume that the higher velocity in the V-shape was induced by the steeper nozzle shape. However, we can also see that although the V-shape has the highest flow acceleration, it does not have the strongest manifestation of helical flows. This is actually seen in the K-form. Thus, it becomes evident that not only the diameter of the narrowest point but also its position within the LVOT has an impact. However, obstruction in HCM is also related to heart rate and loading conditions [1, 9], therefore a flexible phantom could further add knowledge in this matter of interest. The properties of the mixed shape would also need to be measured in a dynamic phantom or in-vivo.

In the past, the LVOT area was assessed using not only CMR but also by 3D echocardiography [9]. It is known that the assessment of LVOT obstruction based on pressure gradient depends on loading conditions leading to fluctuations [2, 3, 6]. CMR combines the advantages of non-invasive and invasive methods. MRI diagnostics are widely used and less dependent on the examiner compared to echocardiography. Non-invasive flow analyses can also be performed, for example, to evaluate obstructions without relying on the fluctuations and indirect determination of the pressure gradient as in echocardiography or the invasive approach of cardiac catheterization. Therefore, CMR is a suitable method for diagnosis and monitoring in patients of all ages. Using our current 3D approach based on time resolved cine-images information about the lengths and shape of the LVOT obstruction could now be added as a control factor. However, there are also notable limitations in reconstructing 3D volumes from 2D slices. The need to hold one’s breath and take successive measurements can lead to shifts between the slices. This is a known issue, but it is tolerated in clinical settings. It is standard practice to analyze functional cardiac parameters from 2D slices [19]. To minimize this phenomenon in our reconstructions of the LVOT (left ventricular outflow tract), multiple checks were performed using anatomical landmarks. When the reconstructed LVOT is viewed in a three-chamber view, there is good alignment of the contours with the anatomical images. It is also worth mentioning that far fewer slices are required for LVOT reconstructions compared to a complete coverage of the entire left ventricle. Thus, a single breath-hold is usually sufficient. Another point to note is that we observed that patients with multiple measurements at different time points (follow-up) showed no changes warranting a different categorization. Nonetheless, expanding the concept to include 3D cine technique would be beneficial. However, this technique is not yet standardized and involves additional components that need to be considered. For instance, the blood-myocardium contrast can be limited without special attention to contrast agent administration, making in-vivo measurements more cumbersome, and insufficient attention to this aspect can complicate the evaluation of LVOT components. Additionally, alignment software is needed, which is still under research. The aforementioned artifacts from breath-holding would be reduced, and trials have shown that measurement times could be decreased [20]. In summary, our current understanding is based on clinical standard measurements and established evaluation techniques, but it is certainly advisable to conduct further analyses using newer measurement methods.

We believe that detailed information about each specific phenotype of obstruction is warranted due to different interventional and surgical therapeutic options being available in patients with HOCM [1]. The interventional approach TASH (transcoronary ablation of septal hypertrophy) depends highly on the anatomy of the septal arteries [1]. The decision making is complex and is primarily based on the localization and type of hypertrophy. The obstruction can also be influenced by the morphology of the mitral valve. There are numerous variations such as longer leaflets or abnormalities in the mitral apparatus [8] guiding the decision towards myectomy. A 3D-model driven surgery is well-known already in other fields of cardiology and cardiothoracic surgery e.g. congenital heart disease [21, 22].

## Conclusion

Three different 3D-shapes of LVOT obstruction in patients with HOCM could be identified. Its hemodynamic profiles as identified by 4D Flow CMR in customized phantoms were different. Future studies are needed to evaluate a potential clinical impact of the different LVOT anatomies. 

## Limitations

Our study has some limitations. The study had a retrospective character in the sense of a pilot study but was intended to identify different shapes of obstruction. Currently the postprocessing is very time consuming and an automatic approach would improve the applicability of our results. Furthermore, the 4D Flow CMR measurements were only applied in phantoms as they were non-existent in the majority of the scans due to its retrospective character. A limitation of our flow measurements is the static character of the phantom because it is well known that the obstruction in HOCM is a dynamic phenomenon, but the intention was to find out if there are different flow profiles at the timepoint of maximum obstruction. For more accuracy, 3D in-vivo measurements of the LVOT including flow measurements would be necessary. This would also prevent possible errors in 3D reconstruction from 2D slices. Also a complete comparison between echo data and CMR was not possible.

## Supplementary Information

Below is the link to the electronic supplementary material.

Supplementary Material 1

Supplementary Material 2

## Data Availability

All data, including the analyses, are stored on the secure study servers of our working group and are available at any time.

## Abbreviations

4D Flow CMR: Three-dimensional (3D) cine (time-resolved) phase-contrast CMR with three-directional velocity encoding
CMR: Cardiovascular magnetic resonance
ES: Endsystolic volume ESV: Endsystolic volume
EDV: Enddiastolic volume
EF: Ejection fraction
HCM: Hypertrophic cardiomyopathy
HOCM: Hypertrophic obstructive cardiomyopathy
LVOT: Left ventricular outflow tract
LV: Left ventricle
NHLBI: National Heart, Lung, and Blood Institute
SAM: Systolic anterior motion
SCMR: Society for Cardiovascular Magnetic Resonance
SSFP: Steady-state free precession
SV: Stroke volume
T: Tesla
